# A comparison on the clinical outcomes of using intraoperative load sensors versus manual balancing in total knee arthroplasty: a systematic review and meta-analysis

**DOI:** 10.1186/s13018-025-06394-8

**Published:** 2025-11-07

**Authors:** Joshua Yeuk Shun Tran, Jenny Wing Lam Lee, Cham Kit Wong, Gloria Yan Ting Lam, Tsz Lung Choi, Wang Fung Rex Mak, Jonathan Patrick Ng, Ki Wai Kevin Ho, Patrick Shu Hang Yung, Michael Tim Yun Ong

**Affiliations:** 1 Chinese University of Hong Kong, Hong Kong SAR, China; 2 Alice Ho Miu Ling Nethersole Hospital, Hong Kong SAR, China; 3 Prince of Wales Hospital, Hong Kong SAR, China; 4 CUHK Medical Centre, Hong Kong SAR, China

**Keywords:** Total knee arthroplasty, Sensor-assisted balancing, Soft tissue balance, Intraoperative load sensors, Randomized controlled trials

## Abstract

**Objectives:**

To compare clinical outcomes and complications between sensor-assisted and manual balancing techniques in total knee arthroplasty, focusing on randomized controlled trials (RCTs).

**Methods:**

A systematic search of the Cochrane Library, Medline, Embase, Scopus, and Web of Science databases was conducted through February 22, 2024. RCTs comparing sensor-guided and manual balancing methods in primary TKA were included. Outcomes assessed include patient reported outcome measures, range of motion, and total complications.

**Results:**

Data from four RCTs (667 knees: 334 sensor-guided, 333 manual) were extracted and analyzed using RevMan V.5.4 with random- and fixed-effects models. The meta-analysis revealed no significant improvement in functional scores for sensor-guided balancing compared to manual techniques (SMD 0.10; 95% CI − 0.15–0.34; I2 = 57%). No significant differences were observed in complication rates (OR 0.84; 95% CI 0.46–1.54; I2 = 0%) or postoperative range of motion (MD 1.20; 95% CI − 0.26–2.65; I2 = 0%).

**Conclusion:**

Sensor-guided balancing did not significantly enhance functional outcomes, reduce complication rates, or improve range of motion compared to manual techniques. While the clinical impact of intraoperative sensors remains limited, they hold promise as training tools to standardize soft tissue balancing. Further high-quality, long-term studies are required to explore their potential benefits and establish definitive guidelines for TKA procedures.

**Supplementary Information:**

The online version contains supplementary material available at 10.1186/s13018-025-06394-8.

## Introduction

Total knee arthroplasty (TKA) has emerged as one of the most commonly performed orthopedic surgeries, providing significant relief for patients with end-stage knee osteoarthritis (OA) [1]. TKA is an effective treatment for end-stage degenerative joint disease, but patient dissatisfaction remains a concern [2]. Complications such as infection, stiffness, and instability can occur, underscoring the necessity for refined surgical techniques to ensure optimal outcomes. It was reported that soft tissue imbalance accounts for 35% of early TKA revisions and often presents clinically as stiffness, instability or tibiofemoral incongruency [3]. A critical aspect of successful TKA is achieving proper alignment and soft tissue balance of the knee joint, which is essential for restoring normal function and longevity of the prosthesis [4].

Gap balancing and soft tissue balancing are fundamental concepts in TKA, as they directly influence joint stability and postoperative function [5]. Proper soft tissue balancing helps ensure that the forces across the knee joint are symmetrical, reducing the risk of complications such as ligament imbalance, which can lead to instability or excessive wear of the prosthetic components [6]. Traditionally, surgeons have relied on various manual techniques to achieve soft tissue balance during TKA. These methods often involve subjective assessments and visual inspections, which can result in variability in surgical outcomes [7]. Manual surgeon-defined assessments have shown poor accuracy in identifying unbalanced knees, with low sensitivity and moderate specificity [7]. Additionally, manual balancing techniques may be influenced by the surgeon's experience and skill level, leading to inconsistent results across different procedures.

In recent years, intraoperative load sensors have gained attention as a technological advancement that could potentially improve soft tissue balancing in (TKA), thereby making ligament balancing less operator-dependent. These sensors provide real-time feedback on joint loading, allowing surgeons to quantify and optimize tibiofemoral forces during surgery [8, 9]. The technology has shown potential benefits, including reductions in gap imbalance, early improvement in patient-reported outcomes, and low rates of arthrofibrosis [9]. Sensor-assisted TKA enables surgeons to analyze various parameters, such as tibiofemoral rotational alignment, compartmental pressures, and prosthetic knee kinematics, potentially leading to improved outcomes and lower revision rates [10].

While the use of sensors intraoperatively achieves improved quantitative soft-tissue balance [11], whether these technical improvements translate into sustained clinical benefits remains controversial. Some research suggests that sensor-assisted TKA can improve soft-tissue balancing and reduce gap imbalance [12], as well as lead to higher rates of improvement in patient-reported outcome measures and range of motion at 6 months post-surgery [13]. Other robust RCTs have found no significant differences in overall survival, satisfaction, or patient-reported outcomes including OKS or FJS at 1 or 2 years within one year of surgery, nor does it reduce reoperation rates [14–16]. Previous analyses question whether a more precisely balanced TKA that is guided by sensor data will ultimately have a significant effect on clinical outcomes [17]. Moreover, there is a lack of a consensus on the definition of soft tissue balance of the knee and the diagnostic accuracy of the reference standard is often unclear [18]. These conflicting findings highlight the need for further research to determine the long-term clinical benefits of sensor-assisted TKA [19].

While an existing meta-analysis that included non-RCTs has been conducted, it introduces potential for selection and confounding bias, which limits the strength of its conclusions. Previous meta-analyses have significant heterogeneity in outcome reporting and a focus on short-term follow-up that is insufficient to assess implant longevity. This systematic review and meta-analysis exclusively of randomized controlled trials (RCTs) aims to clarify the current landscape of research regarding the clinical outcomes associated with intraoperative load sensors compared to manual balancing techniques in TKA to inform clinical practice and guidelines.

## Methods

This meta-analysis adhered to the guidelines outlined in the Preferred Reporting Items for Systematic Reviews and Meta-Analyses (PRISMA), employing both a PRISMA checklist and algorithm [20]. The study was registered with PROSPERO under CRD42024608221 prospectively.

### Search strategy

Cochrane Library, Medline, Embase, Scopus, Web of Science databases were systematically searched until February 22 2024, to identify articles in peer-reviewed journals. The search was performed using the following keywords and their derivatives: “Sensor”, “Load sensor”, “Load measurement”, “Sensor assisted”, “Pressure sensor”, “Sensor guided”, “Sensor balance”, “Introperative sensor”, “Knee arthroplasty,” “Knee replacement,” “Joint replacement,” “Total knee” and “Total joint”. Two independent authors reviewed and screened the search results separately, evaluating them against the eligibility criteria based on their titles and abstracts. Any discrepancies or conflicts were resolved by a third reviewer. A thorough examination of the full-text articles that met the criteria was then carried out, and the references from these articles were manually verified to ensure that all relevant studies were included.

### Eligibility criteria

Inclusion criteria

1. All original randomized controlled trials (RCTs) that report on primary total knee arthroplasty (TKA) indicated for the use of intraoperative load sensors compared to its conventional equivalent.
2. The primary indication for TKA is primary arthritis.
3. English full-text manuscript with available data.
4. Manuscripts with clear outcome measures with attached data presented as or can be transferred to mean and standard deviation values.

Exclusion criteria

1. Studies involving patients with revision TKA.
2. Non-comparative or not reporting outcomes.
3. Review articles, case series, reports, cross sectional and cohort studies.

### Data collection process and data items

A pre-designed data collection sheet in Microsoft Excel was utilized by two independent reviewers to extract data. The collected data items comprised: surname of the first author, year of study, average age of patients, gender ratio of patients, patient count, sensor brand, left to right knee ratio that underwent TKA and post-operative follow-up months.

### Outcomes of interest

The study evaluated several outcomes of interest, with our primary focus being on the functional outcomes assessed by the Knee Society Score (KSS), Knee Society Function Score (KSFS), Knee Injury and Osteoarthritis Outcome Score (KOOS), The Western Ontario and McMaster University score (WOMAC), and Oxford Knee Score (OKS). Our secondary objective was to examine additional factors such as range of motion and total complications.

### Qualitative assessment (risk of bias)

The revised Cochrane risk-of-bias tool for randomized trials (RoB 2) was used in conducting the qualitative analysis [21]. This tool evaluates five key areas: randomization, adherence to intended treatments, missing outcomes, measurement bias, and reporting bias. Two reviewers conducted individual assessments of each study using RoB 2. Subsequently, the third reviewer assessed the final evaluation of each study in collaboration with the reviewers to reach a consensus. To address publication bias, funnel plots were generated using RevMan V.5.4.1

### Quantitative analysis

Meta-analysis was performed using RevMan V.5.4.1(The Cochrane Collaboration, Copenhagen, Denmark). Mean difference and standardized mean difference were measured to analyse continuous variables. Odd ratios with 95% confidence intervals were used to analyse categorical variables. Heterogeneity was measured using I^2^, and results were considered statistically significant at p < 0.05. Funnel plots (Supp Fig. 1–3). were created for all three outcomes of interest. Both fixed-effects and random-effects analysis were performed, and the random-effects model would be presented if there is no funnel plot asymmetry. Random-effects model was used for standardized mean difference, mean difference, and odd ratio. The DerSimonian-Laird method was used for random-effects analysis. The potential sources of heterogeneity were investigated through conducting sub-group analysis, according to the follow-up duration, 6 and 12 months versus 24 months, and the brand of sensor, Verasense Orthosensor.

**Fig. 1:**
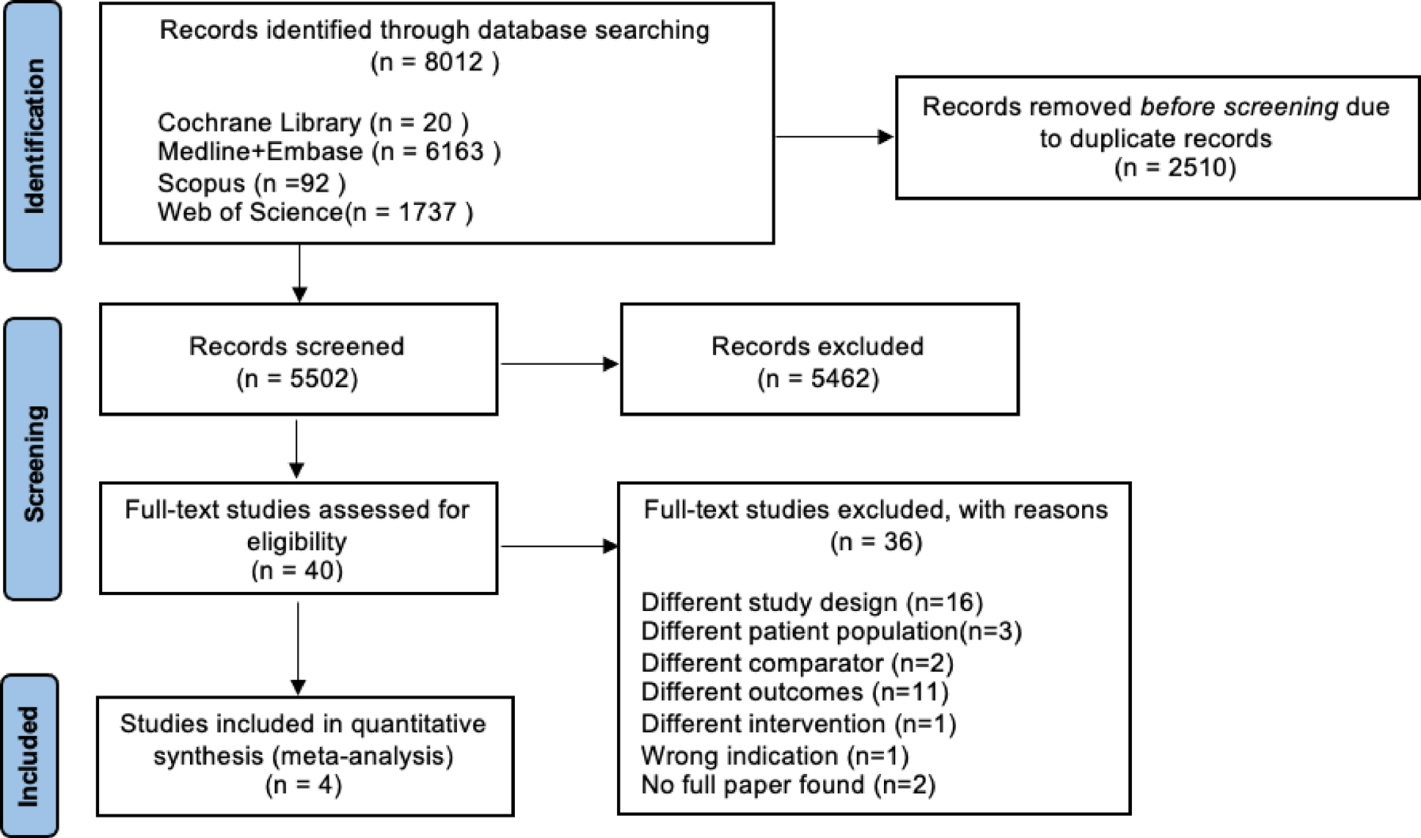
PRISMA flow diagram of included studies

## Results

### Study selection

The initial search yielded 8012 articles, of which 2510 were duplicates and were removed both manually and electronically. The remaining articles were screened by title and abstract, leading to the exclusion of 5462. The full text of the 40 articles that remained were assessed against the inclusion criteria. In the end, four studies met the eligibility criteria and were included into quantitative analysis [22–25]. For studies that reported on approximately the same cohort at different time points, only results from the later study were considered [22, 23, 25]. The PRISMA flowchart illustrating this process is shown in Fig. 1.

### Characteristics of the included studies

The study involved a total of four studies with 667 knees, 334 undergoing sensor-guided knee balancing and the remaining 333 receiving conventional soft tissues balancing. The sample sizes of individual studies ranged from 100 to 285 subjects with mean ages between 67 and 73 years. Three out of four utilized the same sensor guided device (Verasense; Orthosensor, Dania Beach, FL, USA), while the fourth study did not specify the brand of the device used.

All included trials matched their study groups regarding participant age and gender. Results were based on the final follow-up date due to varying follow-up periods. Common outcomes were also compared across studies Table 1.

**Table 1:** Characteristics of included studies

Study	LoE	Age (mean)	Gender	Number of knees	Sensor-guided knee balancing	Manual knee balancing	Follow-up months	Type of sensor
Wood [22]	I	Sensor: 67.1 control: 66.7	M: 69 F: 83	152	76	76	12 months	Verasense, orthosensor
Song [23]	I	Sensor: 72.1 control: 73.0	M: 15 F:85	100	50	50	6 months	Verasense, orthosensor
Sarpong [24]	I	Sensor: 72 control: 70	M: 33 F: 97	130	67	63	24 months	Verasense, orthosensor
Macdessi [25]	I	Sensor: 68.9 control: 69.2	M: 112 F: 168	285	141	144	24 months	

### Quality assessment

All four studies demonstrated a low risk of bias. All studies maintained their groups according to the original randomization, and no study experienced a high dropout rate or failed to report outcomes. A graphic representation of the qualitative assessment can be found in Table 2.

**Table 2:**
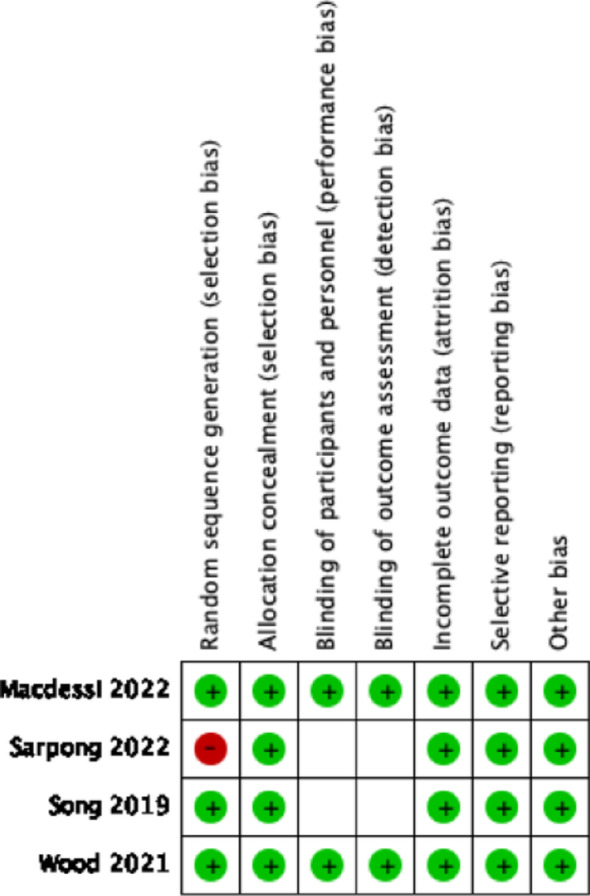
Risk of bias in individual studies

### Functional score (SMD)

Four studies reported on the functional outcomes which were assessed by the Knee Society Score (KSS), Knee Society Function Score (KSFS), Knee Injury and Osteoarthritis Outcome Score (KOOS), The Western Ontario and McMaster University score (WOMAC), and Oxford Knee Score (OKS).

**Fig. 2:**
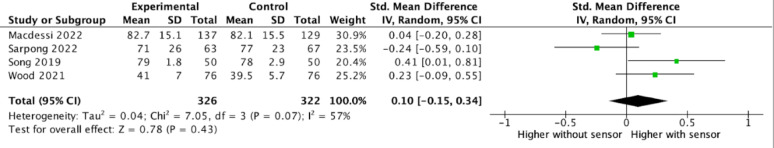
Sensor guided TKA on functional scores

This analysis was conducted at the final follow-up, which ranged from 6 to 24 months. The fixed effect meta-analysis indicated an insignificant improvement in functional scores when TKAs are performed with sensor guided balancing (SMD 0.10; 95% CI − 0.15–0.34) (Fig. 2). The results showed high heterogeneity (I^2^ = 57%, p = 0.07). Further subgroup analysis was done based on follow-up duration. The group with 6 and 12 months (Supp Fig. 4.) showed low heterogeneity (I^2^ = 0%) and significantly improved outcomes (SMD 0.30; 95% CI 0.06–0.55). The group with 24 months (Supp Fig. 5.) showed lower heterogeneity (I^2^ = 42%, P = 0.59) with insignificantly worse outcomes (SMD −0.07; 95% CI −0.35–0.20). Subgroup analysis based on Verasense, Orthosense sensors (Supp Fig. 9.) showed high heterogeneity (I^2^ = 71%, P = 0.51) and insignificantly improved outcomes (SMD 0.13; 95% CI -0.25–0.50).

### Total complications (OR)

**Fig. 3:**
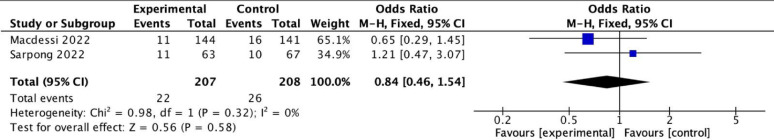
Sensor guided TKA on total complications

Two studies [24, 25] reported on complication events, with respective follow-up periods being 24 months. The included studies at the final follow-up did not report any significant differences between sensor guided knee balancing and manual knee balancing (OR 0.84; 95% CI, 0.46–1.54) (Fig. 3). The results showed low heterogeneity (I^2 ^= 0%). The pooled complications primarily consisted of infection, manipulation under anesthesia, and reoperation. Further subgroup analysis was done for 24 months (Supp Fig. 6.) showing low heterogeneity (I^2^ = 0%) and insignificant differences (OR 0.84; 95% CI 0.46–1.55).

### Range of motion (MD)

**Fig. 4:**
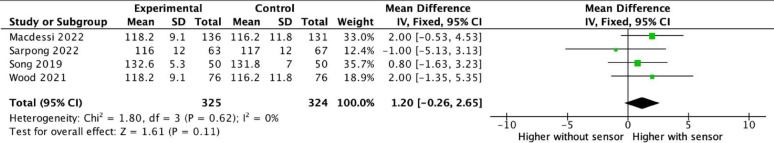
Sensor guided TKA on range of motion

Four studies [22–25] reported on the post-operative range of motion, with their analysis being conducted at the final follow-up, which ranged from 6 to 24 months. The included studies at the final follow-up did not show any significant differences between sensor guided knee balancing and manual knee balancing (MD 1.20; 95% CI − 0.26–2.65) (Fig. 4). The results showed low heterogeneity (I^2^ = 0%). Further subgroup analysis was done based on follo-up duration. The group with 6 and 12 months (Supp Fig. 7.) showed low heterogeneity (I^2^ = 0%) and insignificantly improved outcomes (MD 1.21; 95% CI -0.75–3.18). The group with 24 months (Supp Fig. 8.) showed lower heterogeneity (I^2^ = 32%, P = 0.50) with insignificantly improved outcomes (MD 0.96; 95% CI -1.84–3.76). Subgroup analysis based on Verasense, Orthosense sensors (Supp Fig. 10.) showed low heterogeneity (I^2^ = 0%) and insignificantly improved outcomes (MD 0.80; 95% CI -0.97–2.58).

## Discussion

This meta-analysis demonstrated that sensor-guided balancing in total knee arthroplasty (TKA) does not provide statistically significant improvements in functional outcomes, complication rates, or postoperative range of motion compared with manual balancing. These findings are consistent with existing literature and suggest that, in procedures performed by experienced surgeons, conventional manual balancing remains an effective and reliable approach [26].

Although the pooled findings showed no statistically significant benefit, the clinical interpretation warrants further consideration. The small non-significant improvements observed in functional outcomes (Fig. 2) may indicate that sensor-assisted balancing could help mitigate variability inherent to manual techniques. By providing objective and reproducible intraoperative data, sensors may enhance standardization across institutions and reduce inter-surgeon variability in soft tissue balancing [27]. This is especially relevant in surgical training, where less experienced surgeons can benefit from immediate feedback to develop more consistent balancing techniques.

The minimal differences observed in complication rates (Fig. 3) and postoperative range of motion (Fig. 4) further reinforce that manual balancing, when performed by skilled surgeons, remains highly effective. However, intraoperative sensors uniquely quantify intercompartmental loading, offering a potential framework for refining what constitutes a “balanced knee.” Given the absence of a universally accepted standard [28], sensor data may serve as a foundation for future evidence-based guidelines.

Another explanation for the lack of significant differences lies in the learning curve associated with adopting novel intraoperative technologies [29, 30]. Early integration of sensor systems may initially result in longer operative times and potentially obscure clinical benefits in short-term trials [31]. As the technology matures and surgeons gain proficiency, clearer advantages could emerge. Future research targeting subgroups—such as patients with severe deformities or complex ligament balancing requirements—may better define populations for whom sensor-guided balancing is most beneficial.

The prevalence of knee OA is high, affecting nearly 10 million adults in the United States alone [32]. In Germany, projections indicate a 43% increase in primary TKA incidence by 2050, with the highest growth expected in patients aged 50–65 [33]. Between 2001 and 2010, primary TKA rates in Korea increased by 407%, comparable to rates in Western countries [34]. TKA rates no doubt have increased globally, particularly among younger patients aged 45–64 [32]. With the aging global population and the rising prevalence of obesity that enhances knee joint degeneration, the demand for TKA continues to rise [35]. Despite advancements in disease-modifying antirheumatic drugs, TKA remains an important treatment for rheumatoid arthritis (RA) patients, The annual prevalence of RA in TKA patients remained stable from 2002 to 2013, with a slight increase from 3.31 to 3.50% [36]. Posttraumatic osteoarthritis (PTOA) also accounts for approximately 12% of symptomatic OA cases, with an estimated prevalence of 21.1% in 2022 and projected to reach 40.6% by 2030 [37, 38]. PTOA patients undergoing TKA face higher risks of complications and substantial financial burden, estimated at $3.06 billion annually in the United States [38, 39]. This rising demand presents significant challenges for healthcare systems, necessitating strategies to enhance patient outcomes and manage the increasing burden [40].

This cost implications warrants considerations. Sensor units contribute additional upfront expenses that may not be justifiable given current outcome data. However, if future high-quality studies demonstrate reductions in implant revision rates, improved prosthesis longevity, or cost savings in bundled care models, the investment may be financially sustainable. Indeed, early evidence suggests potential savings within a 90-day postoperative bundle when sensor-guided balancing is incorporated, highlighting the need for robust cost-effectiveness analyses [41].

There are some limitations to the meta-analysis despite the stringent inclusion criteria. First, the different follow-up lengths of the studies introduce inconsistency to the results due to the variable time for patients to recover post-operatively. This may have impacted the functional outcomes of both groups of patients. Moreover, future studies should include longer-term follow-up to show the effects of sensor-guided knee balancing on the durability of knee implants and the revision rates. Second, there is high heterogeneity in the functional outcomes of the patients. One of the reasons is likely due to the combination of various scoring systems into a combined outcome. Other reasons are likely due to the differences in study design that cannot be eliminated. As a result, a random effects model was used to analyse the results. Third, some studies were eliminated as they were non-RCT studies. The exclusion of non-RCTs reduced the overall sample size of the meta-analysis. However, limiting the data aggregation to RCTs only improved the clinical applicability of the results, which fits with the primary focus of the research. Finally, publication bias always exists in systematic reviews and meta-analysis. This is a systemic limitation that cannot be eliminated.

In summary, sensor-guided knee balancing did not demonstrate clear superiority over manual techniques in this meta-analysis. Nonetheless, potential clinical relevance remains in the areas of surgical training, standardization of balancing protocols, and future cost-effectiveness analyses. As the technology matures and further high-quality, long-term trials are conducted, the role of intraoperative sensors in optimizing outcomes for TKA will become clearer.

## Supplementary Information

Below is the link to the electronic supplementary material.

Supplementary Material 1

## Data Availability

No datasets were generated or analysed during the current study.

## Abbreviations

(TKA): Total knee arthroplasty
(OA): Osteoarthritis
(RA): Rheumatoid arthritis
(OA): Posttraumatic osteoarthritis
(PRISMA): Preferred reporting items for systematic reviews and meta-analyses
(RCTs): Randomized controlled trials
(KSS): Knee society score
(KSFS): Knee society function score
(KOOS): Knee injury and osteoarthritis outcome score
(WOMAC): The western ontario and mcmaster university score
(OKS): Oxford knee score
(RoB 2): Cochrane risk-of-bias tool for randomized trials
