# Supplementary material for: A comparison on the clinical outcomes of using intraoperative load sensors versus manual balancing in total knee arthroplasty: a systematic review and meta-analysis
Source: J Orthop Surg Res. 2025 Nov 7;20:970. doi: 10.1186/s13018-025-06394-8 (PMC12595699; doi:10.1186/s13018-025-06394-8)

Supplementary Figure 1. Funnel plot on functional scores


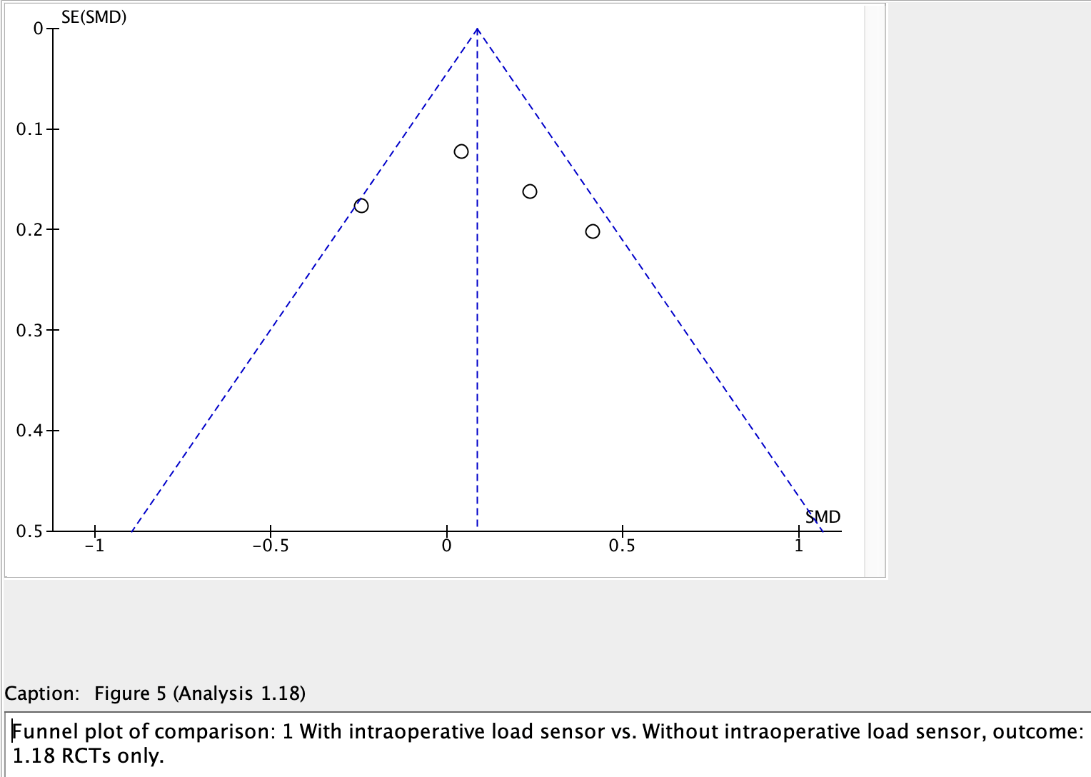


Supplementary Figure 2. Funnel plot on total complications


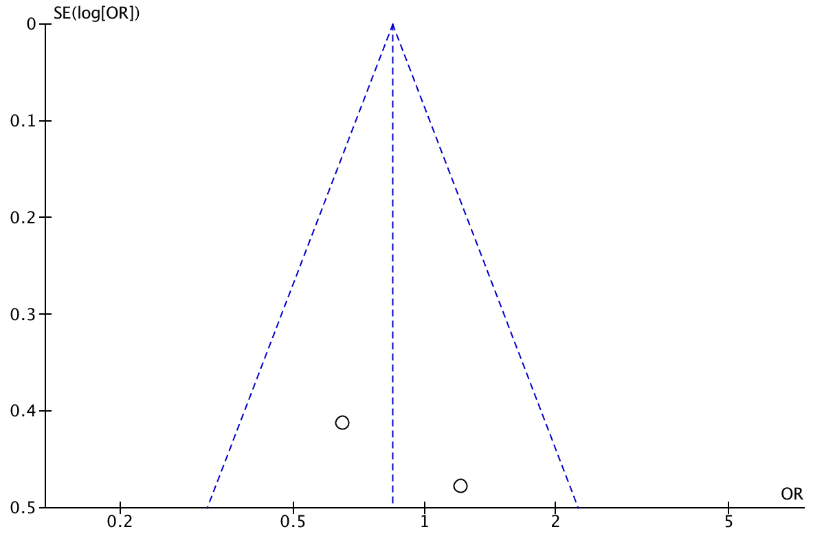


Supplementary Figure 3. Funnel plot on range of motion


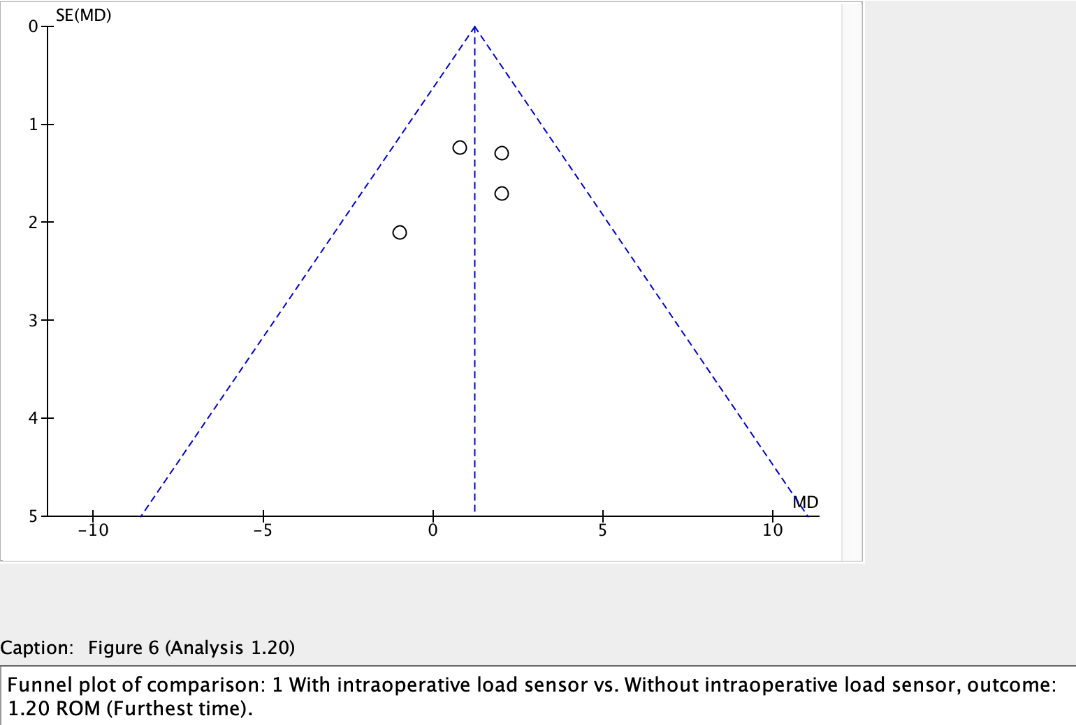


Supplementary Figure 4. Subgroup analysis on functional scores for 6&12 months

*
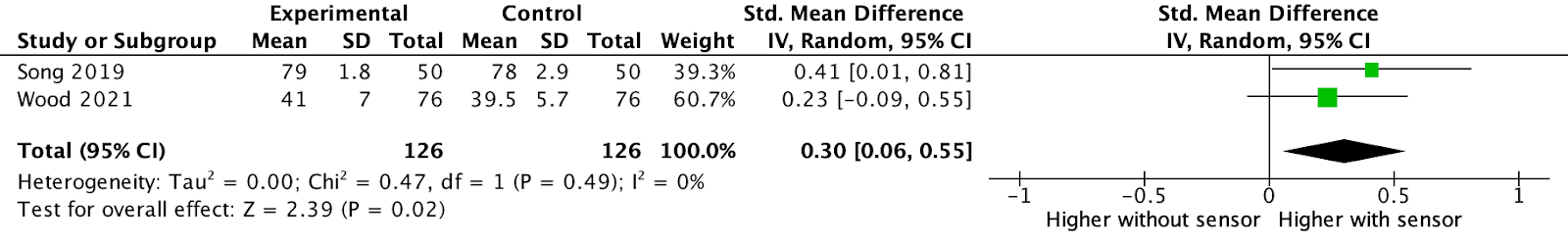
*

Supplementary Figure 5. Subgroup analysis on functional scores for 24 months


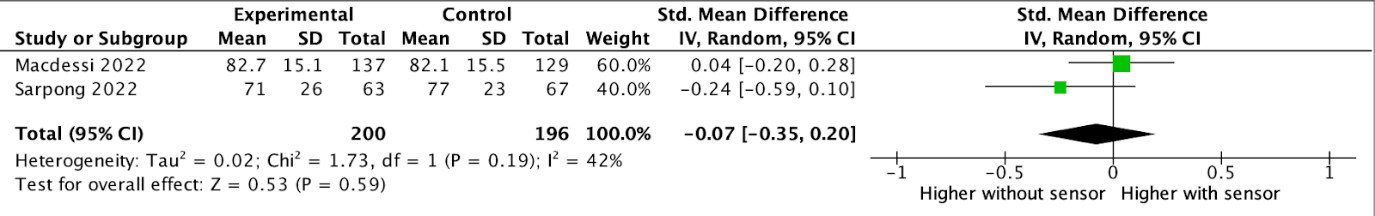


Supplementary Figure 6. Subgroup analysis on total complications for 24 months

*
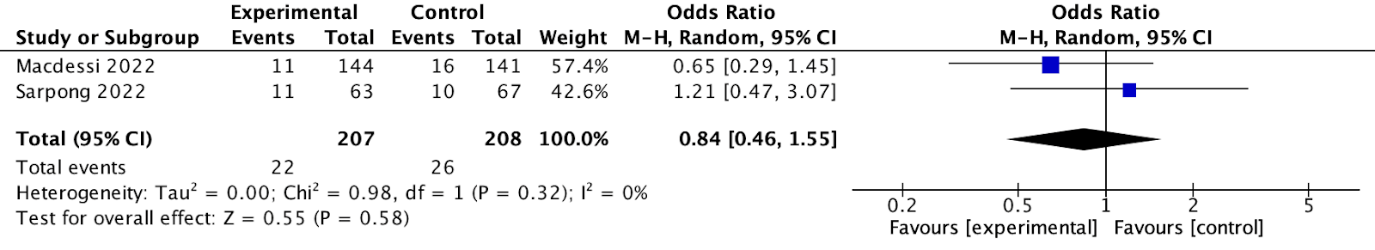
*

Supplementary Figure 7. Subgroup analysis on range of motion for 6&12 months


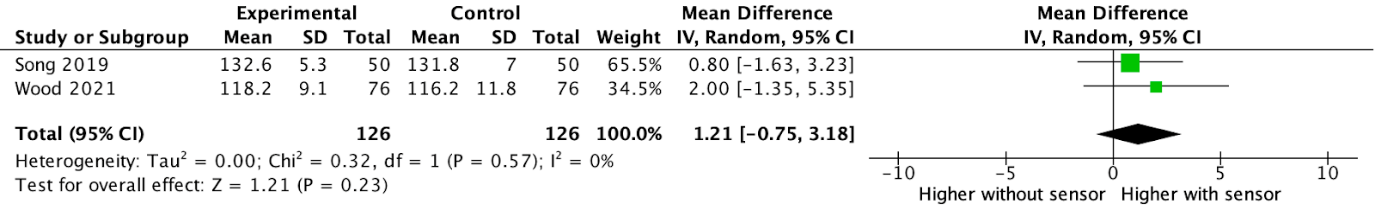


Supplementary Figure 8. Subgroup analysis on range of motion for 24 months


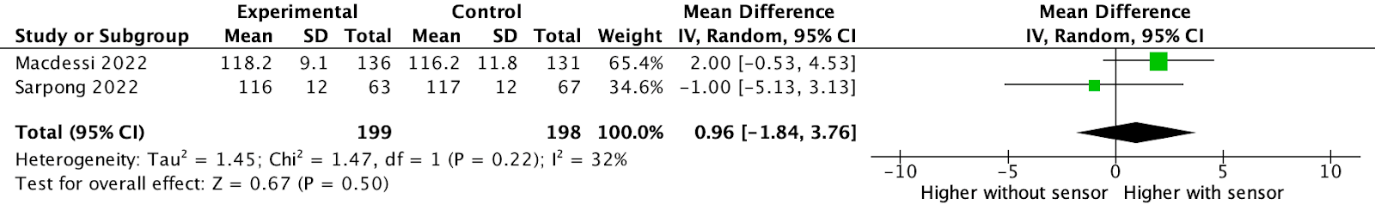


Supplementary Figure 9. Subgroup analysis on functional scores for Verasense, Orthosensor


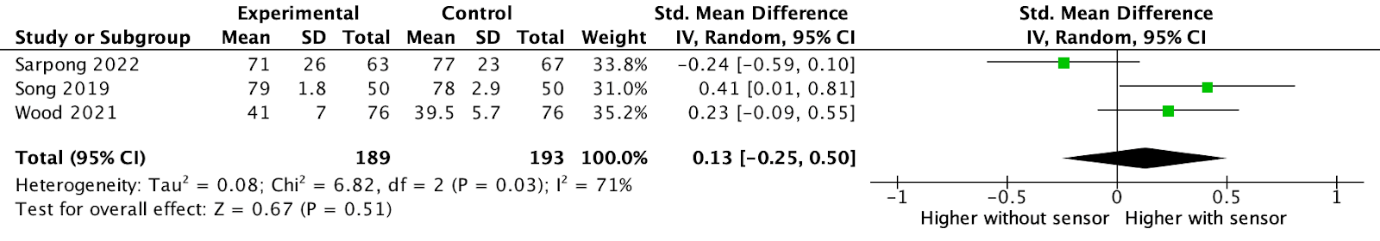


Supplementary Figure 10. Subgroup analysis on range of motion for Verasense, Orthosensor


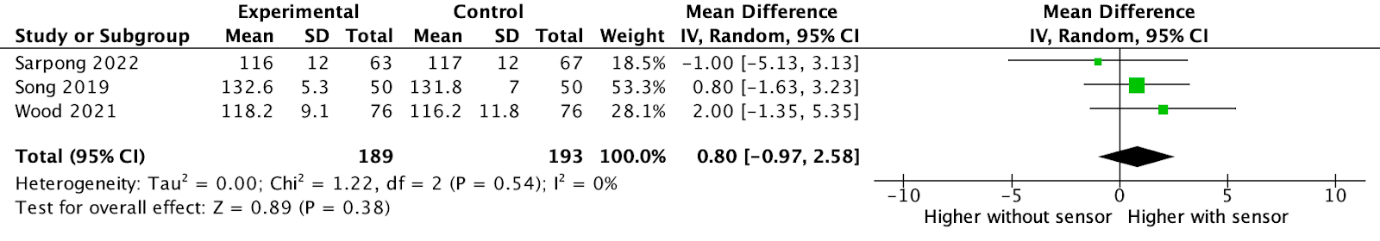

Supplement: Supplementary file 1 — Supplementary Material 1 [file 13018_2025_6394_MOESM1_ESM.docx]
